# Supplementary material for: Macrophages Promote Subtype Conversion and Endocrine Resistance in Breast Cancer
Source: Cancers (Basel). 2024 Feb 5;16(3):678. doi: 10.3390/cancers16030678 (PMC10854660; doi:10.3390/cancers16030678)
Supplement: Supplementary file 1 [file cancers-16-00678-s001.zip › cancers-2825984-supplementary.pdf]

Supplementary Materials

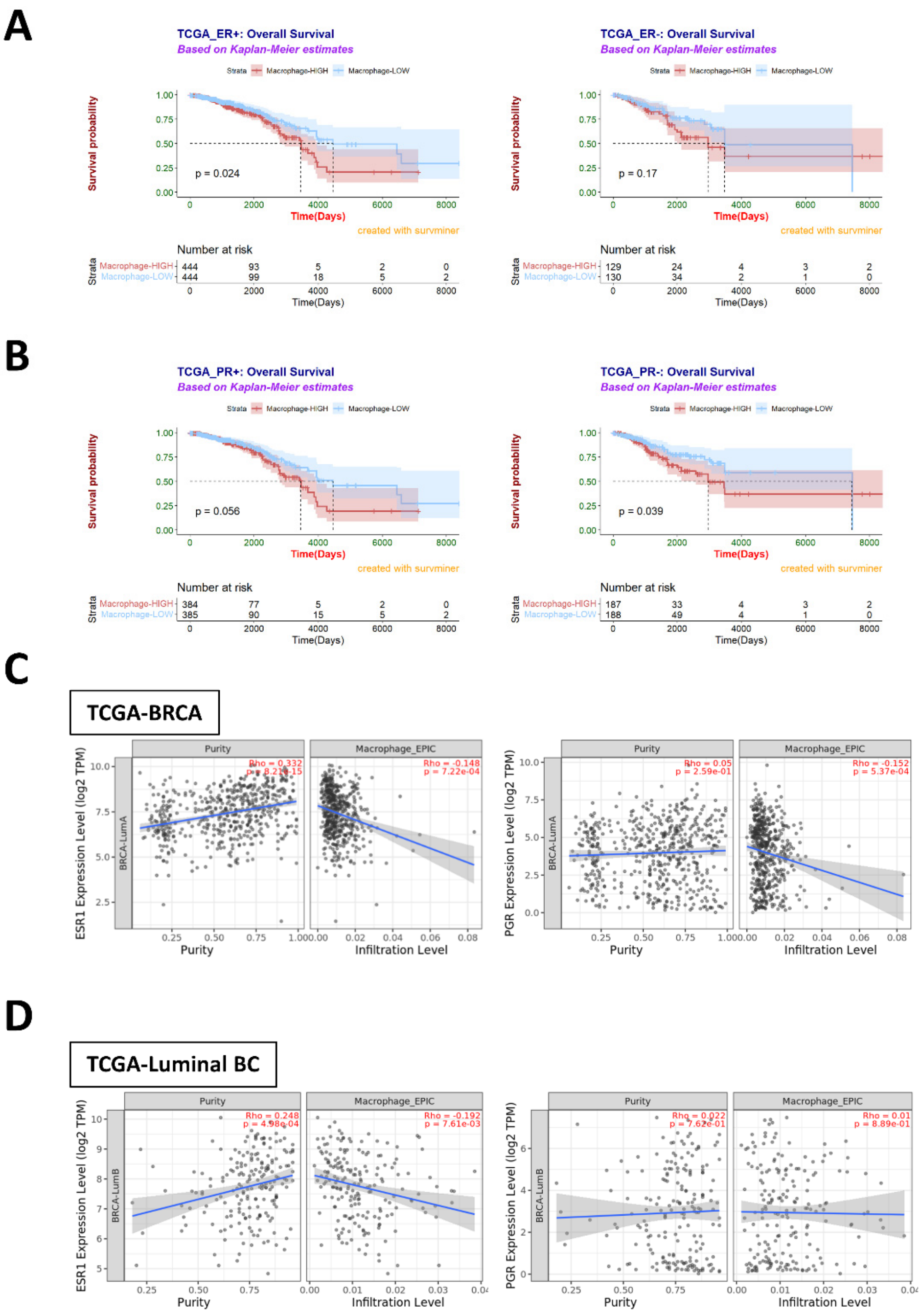

Figure S1. Relationship between molecular classification, lymph node staging, and clinical prognosis. (A, B) Survival analysis of macrophages in ER^{+}, ER^{-}, PR^{+}, and PR^{-} breast cancer, data from TCGA, the infiltration degree of macrophages was grouped using TIMER 2.0. (C, D) Correlation analysis between the infiltration degree of macrophages and the expression of ESR1 in patients with breast cancer or luminal breast cancer in the TCGA dataset, the infiltration degree of macrophages was grouped using EPIC.

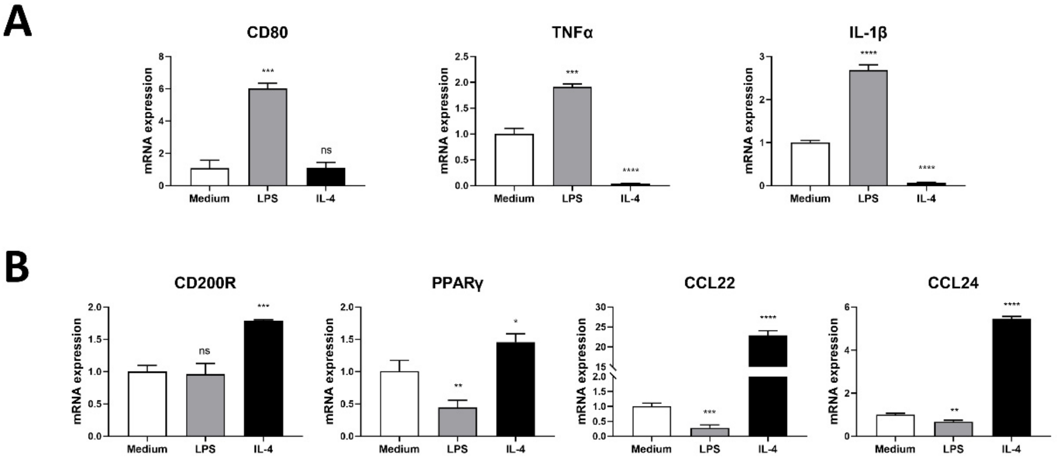

Figure S2. Expression of phenotypic markers on in vitro polarized macrophages.
(A, B) THP-1 were differentiated into macrophages (M0) and polarized in M1 macrophages with LPS (100 ng/ml) and M2 macrophages with IL-4 (20 ng/mL). The phenotypic markers on M1 macrophages were CD80, TNFα, and IL-1β (A). The phenotypic markers on M2 macrophages were CD200R, PPARγ, CCL22, and CCL24 (B). Data present as mean ± SEM, *ns* indicates $p > 0.05$, * $p < 0.05$, ** $p < 0.01$, *** $p < 0.001$, **** $p < 0.0001$.

Supplementary Table S1. Clinical characteristics of the BC tissue microarrays.

Characteristics	No.of patients	Subtype conversion		P-value
		Yes	No	
Patients	47	8	39	
Age				>0.9999
≤60	39	7	32	
>60	8	1	7	
Grade				0.1554
1	7	3	4	
2	26	4	22	
3	9	0	9	
NA	5	1	4	
T classification				0.4060
T1-T2	32	7	25	
T3-T4	15	1	14	
N classification				>0.9999
N0-N1	28	5	23	
N2-N3	19	3	16	
ER				>0.9999
Positive	18	3	15	
Negative	29	5	24	
PR				0.4133
Positive	13	1	12	
Negative	34	7	27	
HER2				0.0574
Positive	20	6	14	
Negative	27	2	25	

Supplementary Table S2. Clinical characteristics of the BC patients.

Characteristics	No.of patients	Subtype conversion		P-value
		Yes	No	
Patients	64	28	36	
Age				>0.9999
≤60	54	24	30	
>60	10	4	6	
Recurrence				>0.9999
NO	61	27	34	
YES	3	1	2	
Stage				0.4898
I-II	18	9	9	
III-IV	35	13	22	
NA	11	6	5	
T classification				0.4119
T1-T2	42	17	25	
T3-T4	18	8	10	
NA	4	3	1	
N classification				0.5948
N0-N1	30	14	16	
N2-N3	29	11	18	
NA	5	3	2	
M classification				0.1685
M0	46	20	26	
M1-M3	13	4	9	
NA	5	4	1	
ER				0.1112
Positive	42	15	27	
Negative	22	13	9	
PR				0.2073
Positive	36	13	23	
Negative	28	15	13	
HER2				0.3134
Positive	25	13	12	
Negative	29	15	24	
Subtype (Primary tumors)				0.1279
Luminal A		9	20	
Luminal B		5	8	
HER2-E		8	4	
TNBC		6	4	

Supplementary Table S3. Primer sequence.

Primer	Sequence（5’ to 3’）
H-MNX1-F	TCCACCGCGGGCATGATCCTG
H-MNX1-R	GGCCCCAGCAGCTCCTCGGCTC
H-NKX2-2-F	CCGGGCCGAGAAAGGTATG
H-NKX2-2-R	GTTTGCCGTCCCTGACCAA
H-β-actin-F	GGCACTCTTCCAGCCTTCCT
H-β-actin-R	GCACTGTGTTGGCGTACAGG
H-ESR1-F	CCCACTCAACAGCGTGTCTC
H-ESR1-R	CGTCGATTATCTGAATTTGGCCT
H-ESR2-F	CAGGCATGCGAGTAACAAGG
H-ESR2-R	GCAGGTCATACACTGGGACC
H-PGR-F	TTATGGTGTCCTTACCTGTGGG
H-PGR-R	GCGGATTTTATCAACGATGCAG

H-EGFR-F	ATCATACGCGGCAGGACCA
H-EGFR-R	TCTGACCGGAGGTCCCAAAC
H-ERBB2-F	TGCAGGGAAACCTGGAACTC
H-ERBB2-R	ACAGGGGTGGTATTGTTCAGC

Uncropped blots for Figure 3

1. T47D

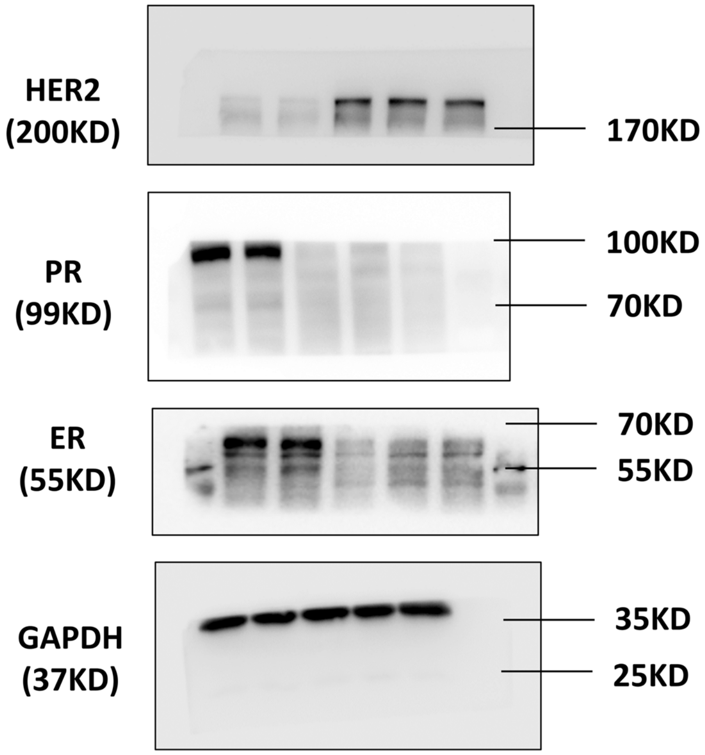

2. BT474

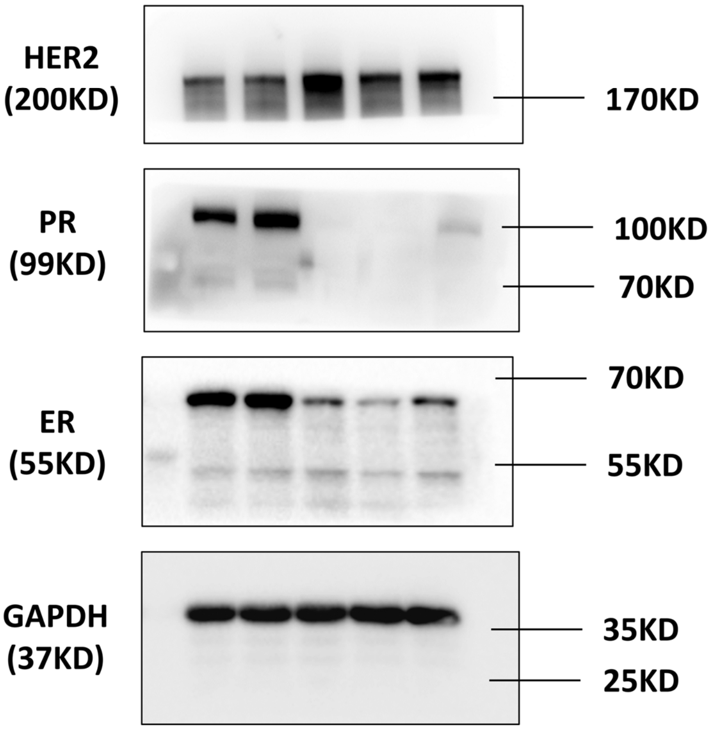

File S1. The original western blot figure.
